# Deep learning-based pancreas volume assessment in individuals with type 1 diabetes

**DOI:** 10.1186/s12880-021-00729-7

**Published:** 2022-01-05

**Authors:** Raphael Roger, Melissa A. Hilmes, Jonathan M. Williams, Daniel J. Moore, Alvin C. Powers, R. Cameron Craddock, John Virostko

**Affiliations:** 1 Department of Diagnostic Medicine, Dell Medical School, University of Texas at Austin, 1701 Trinity St., Stop C0200, Austin, TX 78712 USA; 2 Department of Radiology and Radiological Sciences, Vanderbilt University Medical Center, Nashville, TN USA; 3 Department of Pediatrics, Vanderbilt University Medical Center, Nashville, TN USA; 4 Division of Diabetes, Endocrinology, and Metabolism, Department of Medicine, Vanderbilt University Medical Center, Nashville, TN USA; 5 Department of Pathology, Immunology, and Microbiology, Vanderbilt University, Nashville, TN USA; 6 Department of Molecular Physiology and Biophysics, Vanderbilt University, Nashville, TN USA; 7 VA Tennessee Valley Healthcare System, Nashville, TN USA; 8 Livestrong Cancer Institutes, University of Texas at Austin, Austin, TX USA; 9 Department of Oncology, University of Texas at Austin, Austin, TX USA; 10 Oden Institute for Computational Engineering and Sciences, University of Texas at Austin, Austin, TX USA

**Keywords:** Automatic segmentation, Auto-segmentation, Semantic, T1D, MRI, Neural network, Machine learning, Artificial intelligence, Size

## Abstract

Pancreas volume is reduced in individuals with diabetes and in autoantibody positive individuals at high risk for developing type 1 diabetes (T1D). Studies investigating pancreas volume are underway to assess pancreas volume in large clinical databases and studies, but manual pancreas annotation is time-consuming and subjective, preventing extension to large studies and databases. This study develops deep learning for automated pancreas volume measurement in individuals with diabetes. A convolutional neural network was trained using manual pancreas annotation on 160 abdominal magnetic resonance imaging (MRI) scans from individuals with T1D, controls, or a combination thereof. Models trained using each cohort were then tested on scans of 25 individuals with T1D. Deep learning and manual segmentations of the pancreas displayed high overlap (Dice coefficient = 0.81) and excellent correlation of pancreas volume measurements (R^2^ = 0.94). Correlation was highest when training data included individuals both with and without T1D. The pancreas of individuals with T1D can be automatically segmented to measure pancreas volume. This algorithm can be applied to large imaging datasets to quantify the spectrum of human pancreas volume.

## Introduction

Pancreas volume is reduced in individuals with type 1 and type 2 diabetes [1] and those at risk for T1D [2, 3]. Furthermore, pancreas volume increases with successful therapy in type 2 diabetes [4], suggesting that measurement of pancreas volume may be useful in monitoring diabetes progression and treatment response. However, calculation of the volume of the pancreas currently requires manual segmentation of the pancreas by a trained reader, which is impractical for large clinical trials or studies utilizing large image repositories.

The development of algorithms to automatically segment organs or lesions from medical images has rapidly advanced due to recent breakthroughs in convolutional neural networks and deep learning models [5–7]. A number of studies have segmented the pancreas from abdominal MRI [8, 9] and computerized tomography (CT) scans [10–13]. However, these studies have not included images from individuals with diabetes, where altered pancreas morphology may affect segmentation accuracy. For instance, the pancreas of individuals with diabetes has more irregular borders than individuals without diabetes [4], which may reduce the accuracy of pancreas segmentation approaches trained using only images from non-diabetic individuals. Segmentation of other organs using deep learning, including the brain [14] and inner ear [15], have demonstrated the need to include individuals with pathologies that span the range of anatomical variation in order to improve the generalizability of the segmentation. However, this approach has not been applied to segmentation of the pancreas of individuals with diabetes. In this study, we develop a deep learning-based pancreas segmentation model trained using MRI images from individuals with T1D to enable future studies of pancreas volume in diabetes in large trials and image databases.

## Methods

### Study population

This is a single site retrospective study of previously-acquired abdominal MRI. Study participants were either newly enrolled or part of a previously reported MRI dataset [3] (clinicaltrials.gov identifier NCT03585153). The cohort of MRI scans used for analysis was composed of 185 scans from individuals with T1D and 185 scans from age-matched controls. These studies were approved by the Vanderbilt University Institutional Review Board and performed in accordance with the guidelines and regulations set forth by the Human Research Protections Program.

### Image acquisition and processing

Pancreas MRI was performed on a Philips 3T Achieva scanner (Philips Healthcare, Best, The Netherlands). The image acquisition used for segmentation was a fat-suppressed T2-weighted fast-spin echo sequence with 1.5 × 1.5 × 5.0 mm spatial resolution spanning the pancreas. Each MRI was composed of thirty axial slices with a matrix size of 256 × 256. Imaging was performed in two breath holds with an image acqusition time of 25 s.

A radiologist (M.A.H.), blinded to the diabetes status of each study participant, manually labeled the pancreas on the MRI images to be used as ground truth for segmentation. The network used to automatically segment the pancreas was a 2D U-Net inspired by [10] and [16], where down-convolutions were max pooling layers with size 2 × 2, up-convolutions were transposed convolutions with size 2 × 2 and stride 2, and the final layer was set to one feature channel with a sigmoidal activation function. Each MRI slice was standardized between 0 and 1 to account for a wide range of pixel intensities between MRI scans. The loss function used during the network training was the negative of a smoothed Dice coefficient. The network was trained with Adam optimization at a learning rate of 10^−5^ for 10 epochs and a batch size of one, in agreement with a previous study of pancreas segmentation [10]. Training was implemented in Keras with TensorFlow backend. It took less than one hour to train the network with one GeForce GTX 108 GPU.

Deep learning-based pancreas segmentation was initialized by providing the network with a bounding box encompassing the pancreas, as previously described [10, 13]. We then trained three different models, one using 160 scans of individuals with T1D, one with 160 scans of control individuals, and one with 160 scans equally comprised of scans from controls and patients with T1D (hereafter referred to as the mixed model). The models were trained with four-fold cross validation, with training on 120 out of 160 scans and validation on the remaining 40 scans. For the mixed model, each subset was composed of 20 individuals with and 20 individuals without T1D. We then tested the three models on unseen data composed of 25 individuals with T1D. The segmentation performance was evaluated using volume measurements and the Dice coefficient, which ranges from 0 for no overlap between manual and deep learning-based segmentation to 1 indicating perfect alignment.

### Statistical analysis

Statistical analysis was performed in GraphPad Prism, version 9.2 (San Diego, CA). Differences between independent groups were assessed using unpaired t-test. Linear correlation was assessed by Pearson Correlation Coefficient, with *p* values of 0.05 considered significant. Bland-Altman analysis was performed to assess the difference between deep learning-based and manual measurements of pancreas volume versus the mean volume measurement.

## Results

**Fig. 1:**
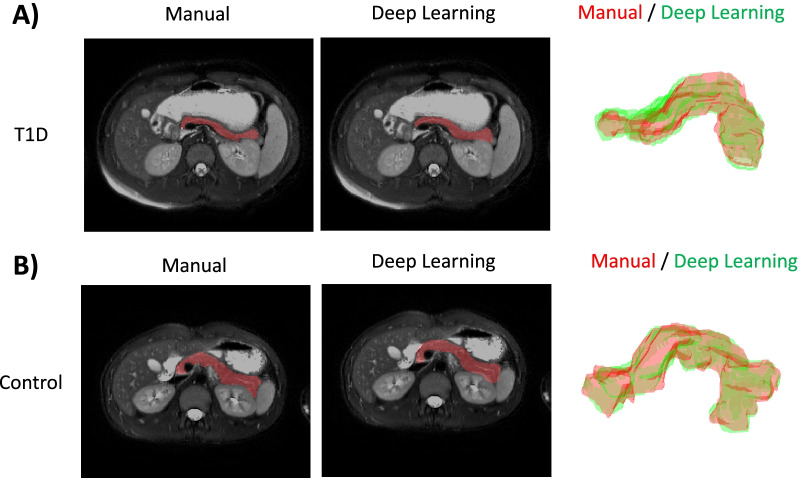
Representative manual and deep learning-based pancreas segmentations from an individual (**A**) with T1D or (**B**) with no pancreas pathology. The representative individual with T1D was a 13-year-old male with 2-month diabetes duration (Dice coefficient = 0.82) while the representative control individual was a 15-year-old male with no known pancreas pathology (Dice coefficient = 0.84). Three dimensional overlays of manual (red) and deep learning-based (green) segmentations are shown for both representative individuals with the pancreas tail oriented to the reader’s left for best visualization. Note the smaller and thinner pancreas size in the individual with T1D

Representative manual and deep learning-based segmentation of the pancreas are shown for an individual with T1D (Fig. 1A) and individual with no pancreas pathology (Fig. 1B). The individual with T1D has a smaller pancreas with a thinner body. Manual pancreas segmentation (left column) displays good agreement with deep learning-based segmentation (middle column) on a representative MRI slice. The three-dimensional pancreas volume constructed from manual segmentation (red) and deep learning-based segmentation (green) display good agreement (right column). Manual and deep learning-based pancreas segmentations displayed a high degree of overlap with mean Dice coefficient of 0.81 ± 0.04 and minimum Dice coefficient of 0.66.

**Fig. 2:**
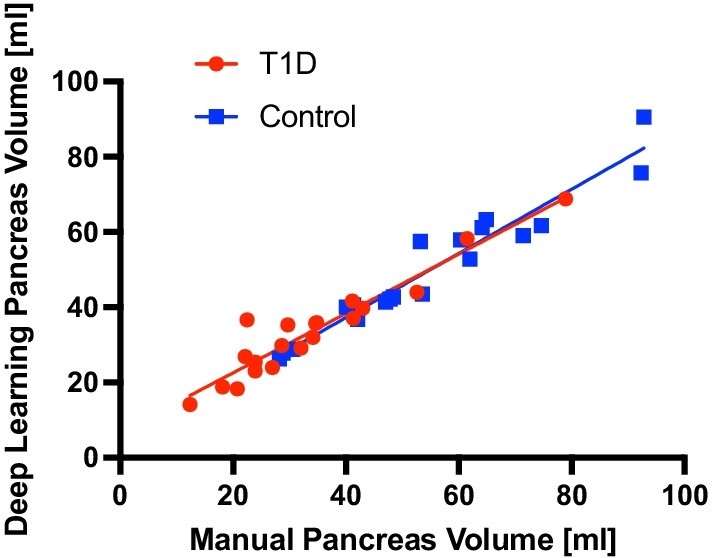
Manual and deep learning-based pancreas volume measurements display correlation across a cohort including individuals with and without T1D (R^2^ = 0.94) and in subsets of individuals with T1D (red; R^2^ = 0.91) or controls (blue; R^2^ = 0.93)

We compared performance of our three models (trained using MRIs from controls, individuals with T1D, and a mixed model incorporating both individuals with and without T1D) on an unseen cohort composed of 25 MRIs from individuals with T1D. The mixed model had a higher Dice coefficient (0.792) and agreement with manually measured pancreas volume (R^2^ = 0.94) than models trained using scans from only control individuals (Dice coefficient= 0.782, R^2^ = 0.91). Manual and deep learning-based pancreas volume measurements derived from the mixed model showed good correlation across a testing cohort of individuals with and without T1D (Fig. 2, R^2^ = 0.94), and in subsets of individuals with T1D (R^2^ = 0.91) or controls (R^2^ = 0.93). Deep learning-based pancreas volume measurements in individuals with T1D were significantly lower than controls (38 ± 12 ml vs. 54 ± 17 ml; *p* < 0.005).

**Fig. 3:**
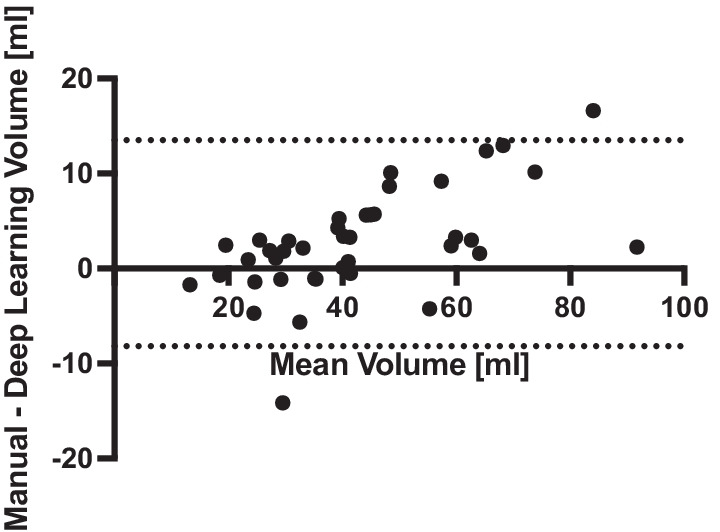
Bland-Altman plot of the agreement between deep learning-based and manual pancreas volume measurements. The 95% limits of agreement are displayed with dotted lines. Deep learning-based measurement of pancreas volume tends to underestimate pancreas size compared with manual measurements (bias = 2.7 ml), particularly at larger pancreas sizes

Bland-Altman analysis was performed to further characterize the agreement between deep learning-based and manual pancreas volume measurements (Fig. 3). Deep learning measurement of pancreas volume tends to underestimate pancreas size compared with manual measurements (bias = 2.7 ml). This underestimation is more pronounced at larger pancreas sizes, as evidenced by a significantly non-zero slope in the Bland-Altman plot (*p* < 0.001). The 95% limits of agreement between deep learning-based and manual pancreas volume measurements are − 8.2 to 13.5 ml.

## Discussion

In this study we applied a neural network to measure pancreas volume in individuals with T1D and demonstrated agreement with manual segmentation by an expert reader. The deep learning-based segmentation calculated smaller pancreas volume in individuals with T1D, in agreement with previous studies using manual segmentation [1–3], but absent the subjectivity inherent to manual segmentation. In fact, the agreement between deep learning and manual pancreas segmentation in this study outperformed the agreement between two different readers performed using images derived from the same study [17]. This finding highlights the subjectivity in manual pancreas segmentations which are in turn used to train deep learning models. The use of images segmented by a single reader is a potential limitation of the study, as our model does not capture the variance induced by multiple readers. However, for large studies in which use of a single reader is not feasible but consistent pancreas segmentation is desired, deep learning-based measurement of pancreas volume can potentially increase reproducibility compared with the use of multiple readers. For instance, longitudinal monitoring of pancreas size in the same individual, which has proven useful in tracking the natural history of T1D [3] and assessment of therapeutic response [4], would benefit from deep learning across assessments as compared with measurements made by different readers at different time points. Pancreas segmentation was improved by training with images from both individuals with and without T1D, as this diverse training set can putatively capture the range of pancreas volume and morphology present in normal and pathological states, as found in brain segmentation [18].

As a small, flexible abdominal organ with a high degree of variation among individuals in both shape and volume, the pancreas is particularly challenging to segment compared with proximal organs such as kidney and liver. This challenge was illustrated in previous automatic segmentation of abdominal organs in which liver, spleen, and kidney segmentation outperformed that of the pancreas [8]. In this study we demonstrate Dice coefficients similar to segmentation performed on a dataset devoid of pancreas pathology [10, 13]. Importantly, when we include both individuals with and without T1D to train the model we observe improved segmentation accuracy, whereas previous pancreas segmentation studies did not include individuals with diabetes. The altered pancreas morphology found in T1D leads to more variation in image features, potentially complicating deep learning-based segmentation. The altered imaging features found in the pancreas in T1D may classify the pancreas of individuals with diabetes, as has been demonstrated in pancreatic cancer [19]. This may prove useful for identifying individuals at risk for T1D or for predicting therapeutic response.

This study is subject to a number of limitations. Bland-Altman analysis demonstrates that pancreas volume tends to be underestimated by deep learning-based segmentation, particularly for large pancreas sizes. Additionally, images were acquired on a single MRI scanner with standardized image acquisition parameters [20]. Deep learning approaches are known to be hampered by difference in MRI scanners and acquisition parameters [21]. Further work is needed to establish pancreas segmentation pipelines incorporating diabetes pathology across multisite data in order to generalize the tool established in this study. Deep learning algorithms for pancreas segmentation are undergoing rapid development and refinement [9–12]. A systematic investigation of segmentation accuracy using different algorithms applied to a common image dataset is needed to compare the performance of these techniques.

## Conclusions

Deep learning-based segmentation of the pancreas can reduce the time and associated cost needed for analysis of pancreas volume and mitigates inter-reader variability. The pancreas segmentation model developed in this study can be applied to large abdominal imaging sets, such as those being acquired as part of the UK Biobank [22], to determine factors which influence pancreas volume and lead to large interindividual variation in pancreas volume.

## Data Availability

The imaging data used and/or analyzed in this study are available from the corresponding author upon reasonable request.

## Abbreviations

T1D: Type 1 diabetes
MRI: Magnetic resonance imaging
CT: Compute tomography
